# Metabolic Pathway of Monounsaturated Lipids Revealed by In-Depth Structural Lipidomics by Mass Spectrometry

**DOI:** 10.34133/research.0087

**Published:** 2023-03-15

**Authors:** Simin Cheng, Donghui Zhang, Jiaxin Feng, Qingyuan Hu, Aolei Tan, Zhuoning Xie, Qinhua Chen, Huimin Huang, Ying Wei, Zheng Ouyang, Xiaoxiao Ma

**Affiliations:** ^1^ State Key Laboratory of Precision Measurement Technology and Instruments, Department of Precision Instrument, Tsinghua University, Beijing 100084, China.; ^2^ Department of Chemistry, Tsinghua University, Beijing 100084, China.; ^3^ Key Laboratory of TCM Clinical Pharmacy, Shenzhen Baoan Authentic TCM Therapy Hospital, Shenzhen, Guangdong 518101, China.; ^4^ Sinopharm Dongfeng General Hospital, Hubei University of Medicine, Experiment center of medicine, Shiyan, Hubei 442008, China.

## Abstract

The study of lipid metabolism relies on the characterization of the lipidome, which is quite complex due to the structure variations of the lipid species. New analytical tools have been developed recently for characterizing fine structures of lipids, with C=C location identification as one of the major improvements. In this study, we studied the lipid metabolism reprograming by analyzing glycerol phospholipid compositions in breast cancer cell lines with structural specification extended to the C=C location level. Inhibition of the lipid desaturase, stearoyl-CoA desaturase 1, increased the proportion of *n*-10 isomers that are produced via an alternative fatty acid desaturase 2 pathway. However, there were different variations of the ratio of *n*-9/*n*-7 isomers in C18:1-containing glycerol phospholipids after stearoyl-CoA desaturase 1 inhibition, showing increased tendency in MCF-7 cells, MDA-MB-468 cells, and BT-474 cells, but decreased tendency in MDA-MB-231 cells. No consistent change of the ratio of *n*-9/*n*-7 isomers was observed in SK-BR-3 cells. This type of heterogeneity in reprogrammed lipid metabolism can be rationalized by considering both lipid desaturation and fatty acid oxidation, highlighting the critical roles of comprehensive lipid analysis in both fundamental and biomedical applications.

## Introduction

Lipids, as an important class of biomolecules, perform critical functions including membrane formation, energy storage, and signal transduction [1–4]. In recent years, our understandings of lipid biofunctions are rapidly increasing in-depth, providing more potential associations between lipids and many diseases, such as cardiovascular disease, COVID-19, and cancer [5–14]. In particular, lipid metabolism reprogramming has been shown to be closely related to cancer cell proliferation, tumor formation, and invasion [12,15,16]. For example, the uptake and de novo synthesis of fatty acid (FA) have been found to be more active in a variety of cancers [17,18]. It has also been reported that a higher degree of lipid unsaturation, reflected by the ratio of stearic acid to oleic acid, is linked to cancer malignancy [19,20]. In addition, phospholipids with longer acyl chains are found to be of higher amounts in squamous cell carcinomas than in normal tissues, and downstream metabolites of FAs such as sphingolipids play an important role in focal adhesion signaling [21,22].

With the rapid development of advanced lipid analysis tools, lipid metabolism reprogramming can be studied with assistance by the information acquired for lipids at more detailed structure levels. In particular, lipid analysis at the C=C location level has become mature with some important demonstrations on its applicability in biological studies [23,24]. The methods developed toward this purpose include ozone-induced dissociation, electron impact excitation of ions from organics, ultraviolet photodissociation, and chemical derivatizations (Paternò–Büchi [PB] reaction, epoxidation reaction, and photooxidation) [25–36]. These tools have not only advanced the lipid structure analysis to an unprecedented level, but also provided opportunities for biomedical analysis, such as discovery of new biomarkers for disease diagnosis [37]. For instance, these methods contributed to the important finding of significant changes in the relative amounts of lipid C=C location isomers in blood or tissue samples for cancer diseases, such as human prostate cancer, colorectal cancer, breast cancer, lung cancer, and lymph nodes with thyroid cancer metastasis [8,32,38–41]. These new findings enabled by the new analytical capability led to further investigation on the underlying biological mechanisms leading to such changes in lipid composition. For instance, the fatty acid desaturase 2 (FADS2) pathway, an alternative FA desaturation pathway bypassing stearoyl-CoA desaturase 1 (SCD1), can cause an abnormal and elevated amount of *n*-10 isomers, adding to cancer plasticity [7,23,24]. Such kind of studies can enhance our understanding of the altered lipid metabolisms related to cancers and is important for developing more effective cancer therapies.

**Fig. 1.**
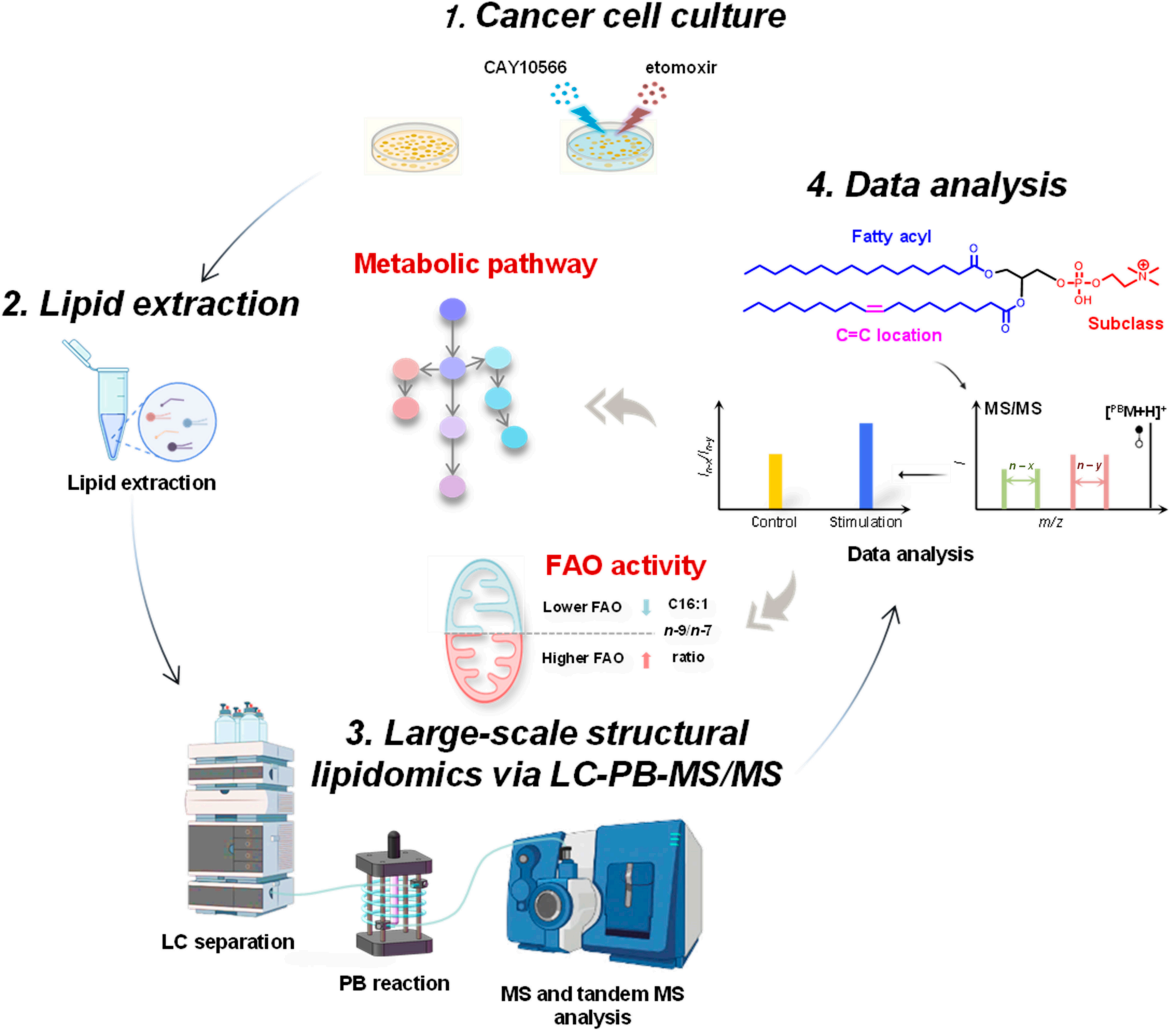
Deep GP structure analysis workflow by LC-MS coupling with online PB reaction to reveal C=C location isomer metabolic pathway variations and FAO activity.

In this work, we aim to study the underlying mechanism of altered lipid compositions, particularly at the C=C location level, after modulating the lipid enzymes’ bioactivity (Fig. 1). The newly established PB derivatization coupled with liquid chromatography–tandem mass spectrometry (LC-PB-MS/MS) workflow was used to monitor omics-level lipid alterations. Upon SCD1 inhibition, lipid unsaturation in human breast cancer cells decreased significantly, as monitored by lipid profiling. Using the new analytical tools, we quantified the relative amounts of the C=C location isomers, including *n*-10, *n*-9, and *n*-7 series isomers. A comprehensive analysis of C=C location isomers toward different breast cancer cell lines revealed that both lipid dehydrogenation and lipid oxidation coregulated lipid fine structure metabolic reprogramming. Interestingly, we have found that the C16:1 *n*-9/*n*-7 isomer ratio, which is highly correlated with fatty acid oxidation (FAO) activity, was closely related to the invasiveness of breast cancer cells. Our research demonstrates that lipidomics can be a powerful tool to explore the underlying mechanisms of lipid reprogramming in diseased states. Structural lipidomics will therefore not only serve as a more cost-effective analytical method to acquire detailed lipid composition but also contribute to the study of lipid metabolism.

## Results

### Monitoring lipid unsaturation after SCD1 inhibition

**Fig. 2.**
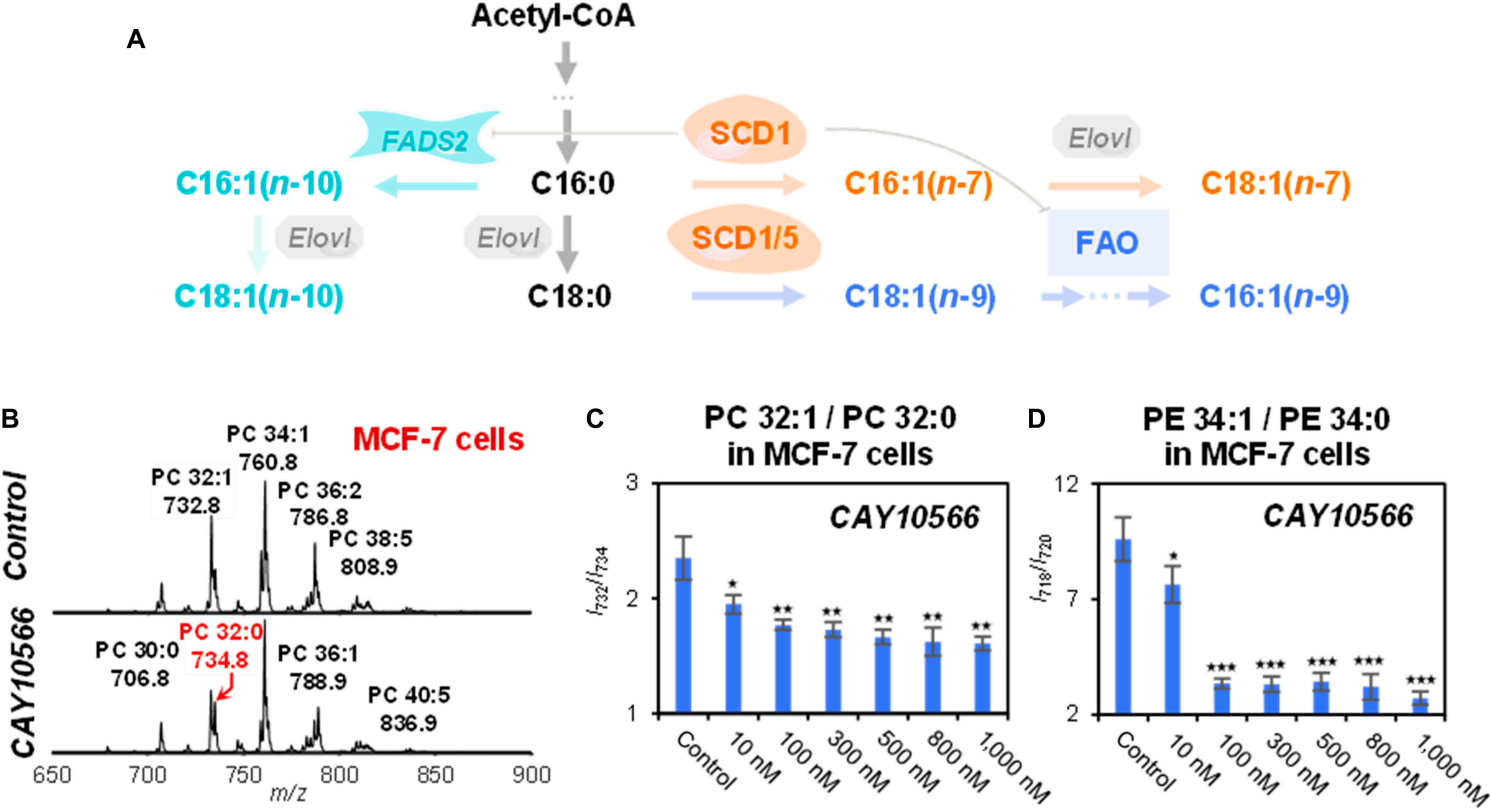
Lipid unsaturation decreased upon SCD1 inhibition. (A) Biosynthetic pathways of monounsaturated FA. SCD1 and SCD5, stearoyl-CoA desaturase 1 and 5; FADS2, fatty acid desaturase 2; FAO, fatty acid oxidation; Elovl, fatty acid elongase. (B) Mass spectra of PC profile before and after SCD1 inhibition in MCF-7 cells. (C and D) The relative ratios of PC 32:1/PC 32:0 and PE 34:1/PE 34:0. Differences between CAY10566-treated group samples and control group samples were evaluated for statistical significance using the 2-tailed Student’s *t*-test (*P < 0.05, **P < 0.01, ***P < 0.001). Error bar represents the standard deviation, *n* = 3.

The de novo biosynthesis of monounsaturated lipids has been well studied, as shown in Fig. 2A. The C=C location isomers of C18:1- and C16:1-containing glycerol phospholipids (GPs) consist of *n*-10, *n*-7, and *n*-9 isomers. In this lipid metabolic network, SCD1 serves as a central role and is responsible to produce monounsaturated lipids. The composition of unsaturated lipids frequently undergoes significant metabolic changes in various diseased states, due to altered activity of SCD1 and related enzymes. We show in this section the changes in the lipidome of MCF-7 human breast cancer cells after SCD1 inhibition (*via* CAY10566). Overall, the degree of unsaturation in the lipidome of MCF-7 cells decreased after SCD1 inhibition, consistent with previous studies [42–44]. Specifically, the relative intensity of phosphatidylcholine (PC) 32:0 increased while the intensity ratio of PC 32:1(*m*/*z* 732) to PC 32:0 (*m*/*z* 734) decreased as SCD1 inhibitor concentration increased (Fig. 2B, C). A similar trend was observed for phosphatidylethanolamine (PE) (Fig. S1), and the intensity ratio of PE 34:1(*m*/*z* 718) to PE 34:0 (*m*/*z* 720) decreased to reach a plateau at 100 nM CAY10566 (Fig. 2D). The inhibition of SCD1 blocked the dehydrogenation of C16:0 and C18:0, resulting in the accumulation of C16:0 and C18:0. As a result, the abundance of PC 32:0 and PE 34:0 increased. Such higher levels of saturated lipids indicate a more rigid cell membrane and low membrane fluidity [45,46]. Other than lipid desaturation, we also observed altered cell morphology and a reduced number of growing cells. The pseudopod extension was clearly observed, possibly due to reduced cell viability and proliferation after SCD1 inhibition (Fig. S2A). The cell viability assay showed a significant inhibition effect on cell proliferation by CAY10566 in vitro (Fig. S2B), suggesting the essential role of SCD1 for cell growth and survival.

### Increase of *n*-10 isomers in C16:1- and C18:1-containing GPs after SCD1 inhibition

**Fig. 3.**
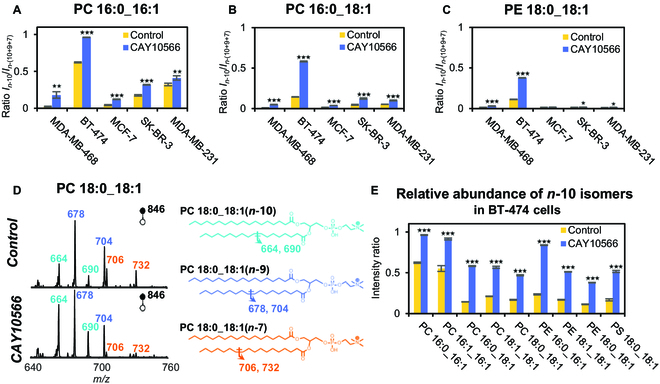
The relative amount of *n*-10 C=C location isomers increased significantly after SCD1 inhibition. (A to C) Relative increase of *n*-10 isomers in C16:1- and C18:1-containing GPs after SCD1 inhibition. (D) PB-MS/MS spectra of PC 36:1 before and after SCD1 inhibition in BT-474 cells and the corresponding structures. (E) Compositional increase of *n*-10 C=C location isomers in BT-474 cells after SCD1 inhibition. Differences between the 2 groups were evaluated for statistical significance using the 2-tailed Student’s *t* test (*P < 0.05, **P < 0.01, ***P < 0.001). Error bar represents the standard deviation, *n* = 3.

Recently, scientists have taken a new look at FADS2, also known as D6D, which was exploited to produce *n*-10 isomers in carcinoma, illustrating heterogeneity in FA desaturation and making it more plastic in signaling networks [7,23]. Herein, we studied the relative amounts of *n*-10 isomers after SCD1 inhibition in breast cancer cells. As expected, the relative amounts of *n*-10 isomers in both C16:1- and C18:1-containing GPs increased after SCD1 inhibition (Fig. 3A to C). For example, in BT-474 cells, the *n*-10 isomer for PC 18:0_18:1 increased significantly (Fig. 3D), as evidenced by the increased abundance of diagnostic ions (*m*/*z* 664 and *m*/*z* 690) for C=C location isomers. The above observations can be rationalized by suppressed *n*-9 and *n*-7 isomers synthesis and possibly increased FADS2 activity upon SCD1 inhibition [7]. The increased amounts of polyunsaturated GPs after SCD1 inhibition, whose biosynthesis is also closely related to FADS2, provide additional evidence to support increased FADS2 activity (Fig. 2B and Fig. S1).

It is worth noting that BT-474 cells contain much higher levels of *n*-10 isomers than other breast cancer cell types and the lipid isomeric changes are also more significant after SCD1 inhibition. We performed LC-PB-(−MS3) in negative ion mode for the direct characterization of unsaturated fatty acyl isomers (Fig. S3), confirming the changes of *n*-10 isomers in BT-474 cells observed by LC-PB-(+MS2). Global profiling of GPs in BT-474 cells following CAY10566 inhibition shows consistent increases of *n*-10 isomers for all GP types, including PCs, PEs, or phosphatidylserines (PSs) (Fig. 3E). The relative expression levels of both SCD1 and FADS2 were analyzed by using basal genome-wide expression data previously collected from shared stocks on the Cancer Cell Line Encyclopedia (CCLE) database (http://www.broadinstitute.org/ccle/home, Fig. S4). Compared with SCD1, FADS2 expression generally agrees well with our C=C specific lipid analysis, except for SK-BR-3 cells. For BT-474 cells, in particular, it has the highest level of FADS2 expression and the highest relative amounts of n-10 isomers, as expected.

### Global profiling of *n*-7 and *n*-9 isomers in C18:1-containing GPs after SCD1 inhibition in MCF-7 cells

**Fig. 4.**
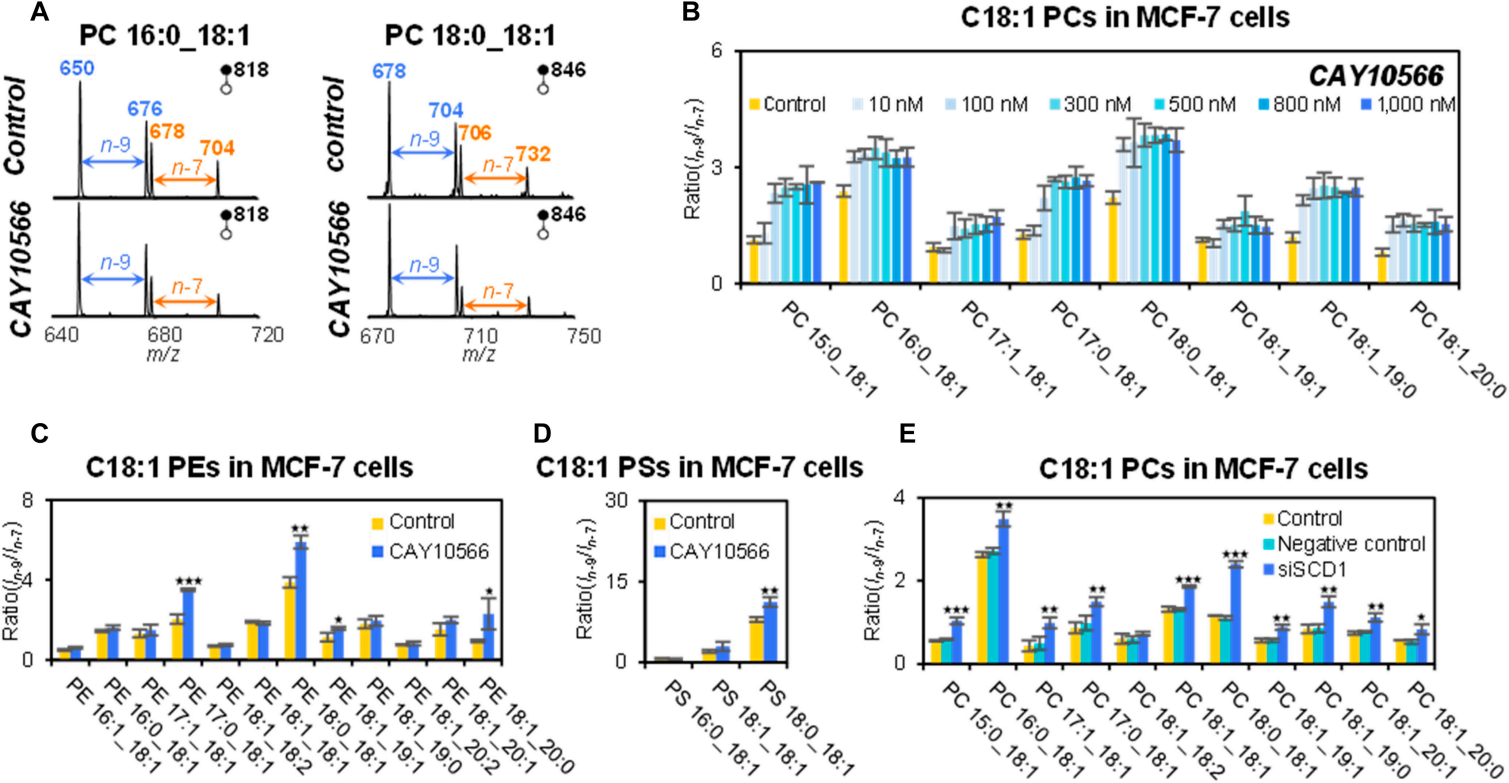
Isomer-resolved lipidomics analysis of C18:1-containing GPs in MCF-7 cells. (A) PB-MS/MS analysis of PC 34:1 and PC 36:1 before and after SCD1 inhibition. (B to D) Compositional variations of C=C location isomers in C18:1 fatty acyl in PCs, PEs, and PSs after SCD1 inhibition. (E) Compositional variations of C=C location isomers in C18:1-containing PCs after siRNA-mediated gene silencing of SCD1. Differences between the 2 groups were evaluated for statistical significance using the 2-tailed Student’s *t* test (*P < 0.05, **P < 0.01, ***P < 0.001). Error bar represents the standard deviation, *n* = 3.

The most common and abundant GPs with C=C location isomers are *n*-7 and *n*-9 isomers in C18:1-containing GPs. The isomeric compositions of C18:1 GPs showed significant changes after SCD1 inhibition, along with decreased lipid desaturation as revealed by systematic LC-PB-MS/MS analysis. The typical MS/MS spectra of PC 16:0_18:1 and PC 18:0_18:1 in MCF-7 cells before and after SCD1 inhibition are presented in Fig. 4A. Clearly, the intensity ratios of *n*-9/*n*-7 isomers for both PCs increased after SCD1 inhibition. The increased intensity ratios of *n*-9/*n*-7 isomers for other C18:1-containing PCs, PEs, and PSs are shown in Fig. 4B to D, consistent with previous studies [32,47]. Similar experimental results were observed after siRNA-mediated gene silencing of SCD1, in which the ratios of *n*-9/*n*-7 isomers increased in both C18:1-containing PCs, PEs, and PSs (Fig. 4E and Fig. S5A and B). Additionally, gene silencing of SCD1 by siRNA reduced SCD1 mRNA expression levels in MCF-7 cells (Fig. S5c). This set of experiments verifies the known effects of lipid desaturases to modulate lipid desaturation and lipid C=C location isomer composition. From the perspective of biosynthetic pathways in human, there are 2 isoforms of stearoyl-CoA desaturases, i.e., SCD1 and SCD5 [48], both of which catalyze the desaturation of C18:0-CoA to produce C18:1 (*n*-9)-CoA. However, only SCD1 can convert C16:0-CoA to C16:1 (*n*-9)-CoA, which can be further elongated to C18:1 (*n*-7)-CoA. Therefore, once SCD1 is inhibited, the pathway leading to the generation of C18:1 (*n*-7)-CoA is more severely blocked while C18:1 (*n*-9)-CoA can still be produced via SCD5, leading to increased *n*-9/*n*-7 isomer ratios for C18:1 GPs. Moreover, changes in the chain compositions of GPs were observed by LC-MS/MS in negative ion mode. For instance, upon increased CAY10566 treatment, the relative amount of PC 18:1_18:2 decreased and that of PC 16:0_20:3 increased, due to suppressed C18:1-CoA synthesis by the inhibition of lipid desaturation (Fig. S6).

### Differential lipidomics response to SCD1 inhibition in human breast cancer cells

**Fig. 5.**
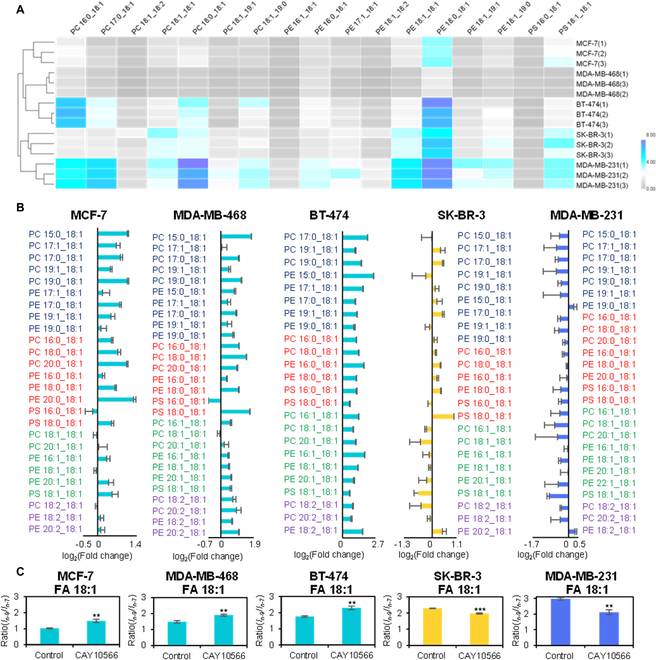
Human breast cancer cell lines responded differently to SCD1 inhibition. (A) Hierarchical cluster analysis discriminated the 5 subtypes of human breast cancer cells by quantitative analysis of *n*-9/*n*-7 isomers in C18:1-containing GPs. (B) Fold change of *n*-9/*n*-7 isomeric ratio in C18:1-containing GPs after SCD1 inhibition. (C) Compositional variations of C=C location isomers in FA 18:1 after SCD1 inhibition. Differences between the 2 groups were evaluated for statistical significance using the 2-tailed Student’s *t* test (*P < 0.05, **P < 0.01, ***P < 0.001). Error bar represents the standard deviation, *n* = 3.

It has been reported that lipid metabolism among different breast cancer cell lines is quite different, but few in-depth studies are performed at the C=C location level with large-scale lipidomics [32,49–51]. We conducted a comprehensive lipidomics mapping of C18:1-containing GPs and distinct differences among different subtypes of human breast cancer cell lines. The observed compositions of GPs C=C location isomers can enable the discrimination of different breast cancer cell subtypes via hierarchical cluster analysis (Fig. 5A), in which the cell lines of SK-BR-3 and MDA-MB-231 show a higher degree of similarity with a much high intensity ratio of *n*-9/*n*-7 isomers in C18:1-containing GPs.

Different from consistent increases in *n*-10 GP isomers, breast cancer cells of different subtypes show entirely different sensitivity to SCD1 inhibition with diverse tendencies of the ratios of *n*-9/*n*-7 isomers in C18:1-containing GPs. The fold changes of C=C location isomers in C18:1-containing GPs after SCD1 inhibition are shown in Fig. 5B. As expected, the intensity ratios of *n*-9/*n*-7 isomers of most C18:1-containing GPs in MDA-MB-468 and BT-474 cells increased, similar to MCF-7 cells. However, SK-BR-3 cells and MDA-MB-231 cells showed distinct changes in terms of *n*-9/*n*-7 isomer ratios. For SK-BR-3 cells, the *n*-9/*n*-7 isomer ratios increased in monounsaturated GPs but decreased in polyunsaturated GPs. For MDA-MB-231 cells, the *n*-9/*n*-7 isomer ratios decreased in both monounsaturated and polyunsaturated GPs, except for few GP species, e.g., PE 18:1_18:2 and PE 18:1_19:0. We divide all C18:1-containing GPs into 4 groups based on their fatty acyl compositions that include an odd-number fatty acyl, a saturated fatty acyl, a monounsaturated fatty acyl, or a polyunsaturated fatty acyl. In each group, the changes in *n*-9/*n*-7 isomer ratios are overall consistent, possibly suggesting a role of the other fatty acyl on the selective incorporation of C18:1 *n*-9/*n*-7 into the GP [8].

The different changes of *n*-9/*n*-7 isomers of C18:1-containing GPs in breast cancer cell lines aroused our interest in discerning C=C locations in FA building blocks. As displayed in Fig. 5C, the ratio of *n*-9/*n*-7 isomers in free FA 18:1 in MCF-7 cells, MDA-MB-468 cells, and BT-474 cells showed increased variation trends, and that in MDA-MB-231 cells showed decreased variation trends, corresponding to its GP tendency. Because of 2 trends in the change of phospholipids, the change of FA 18:1 in SK-BR-3 cells is the most unpredictable. The final result showed a declining process. Combined together, these variations in *n*-9/*n*-7 isomers of C18:1-containing GPs suggested that there must be some other factors that influence the C=C location isomeric ratio, and the universality of drugs targeting SCD1 treatment should be well considered since different cancer cell subtypes respond differently even within the same cancer type.

### FAO also modulates the composition of GP C=C location isomers

**Fig. 6.**
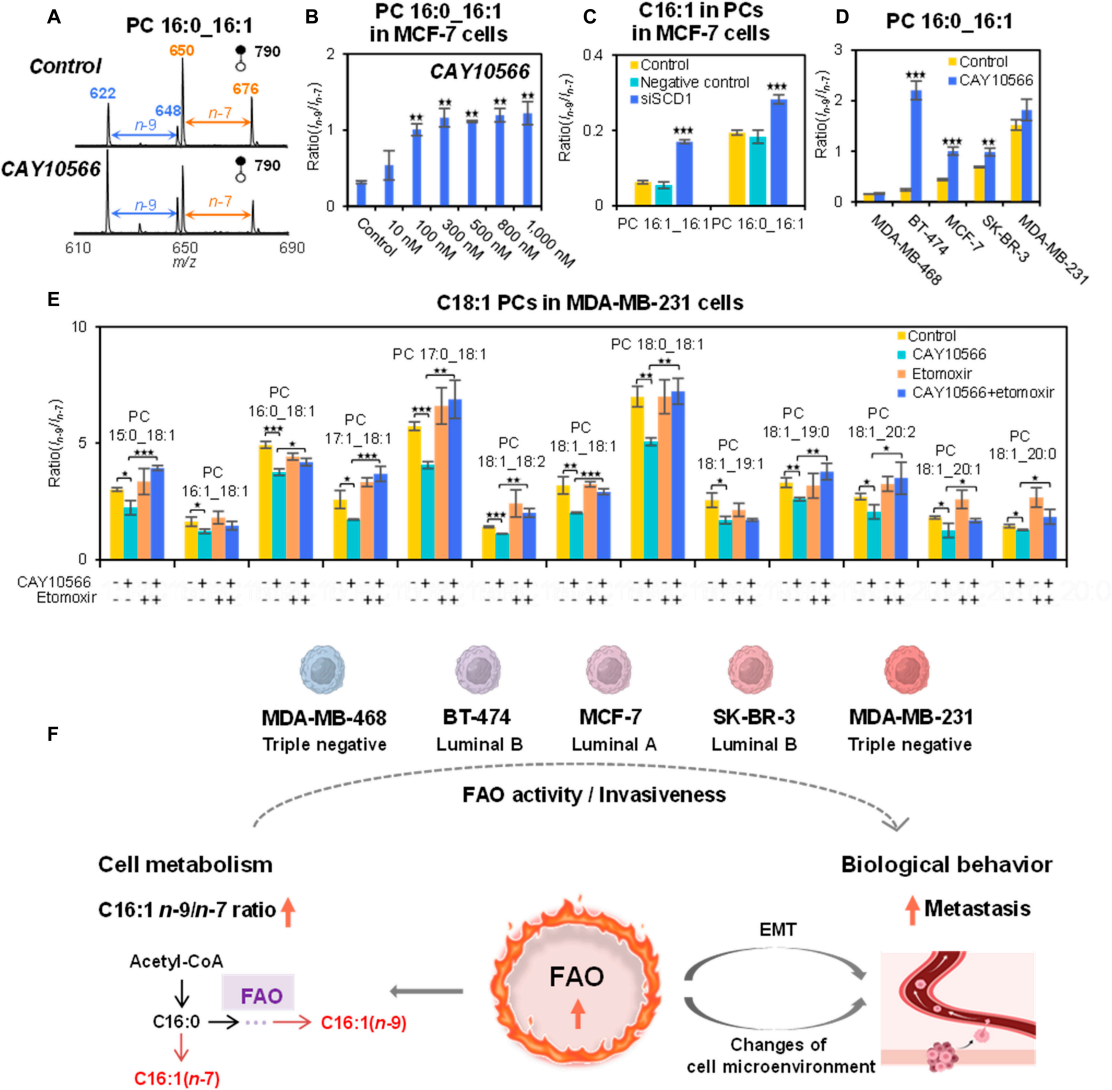
The measurement of FAO reveals its potential relationship with breast cancer cell line invasiveness. (A) PB-MS/MS spectra of PC 16:0_16:1 in MCF-7 cells before and after SCD1 inhibition. (B to D) Compositional variations of C=C location isomers in C16:1-containing GPs after small-molecule inhibitor or siRNA-mediated gene silencing of SCD1. (E) The inhibition of FAO by etomoxir compensates for the decrease of *n*-9/*n*-7 isomers in C18:1-containing GPs caused by SCD1 inhibition in MDA-MB-231 cells. (F) Schematic illustration of the indicators to measure FAO activity, and the relationship between FAO and metastasis. The indicators of C16:1 *n*-9/*n*-7 ratio can be used to infer FAO activity and cancer cell invasiveness. Differences between the group samples were evaluated for statistical significance using the 2-tailed Student’s *t* test (*P < 0.05, **P < 0.01, ***P < 0.001). Error bar represents the standard deviation, *n* = 3.

Based on the biosynthetic pathway of different lipid C=C location isomers (Fig. 2A), downstream of the SCD1-catalyzed lipid desaturation, C16:1 (*n*-9)-CoA is synthesized from C18:1 (*n*-9)-CoA via FAO. Therefore, we hypothesize that FAO may contribute to the regulation of C18:1 (*n*-9) and C18:1 (*n*-7). Then, we analyzed PC 16:0_16:1, which is the most abundant GPs with C16:1 (Fig. S7). Coupled with PB reaction, 2 pairs of C=C location specific diagnostic ions were detected for PC 16:0_16:1, indicating the presence of *n*-9 (*m*/*z* 622 and 648) to *n*-7 (*m*/*z* 650 and 676) isomers (Fig. 6A).

For MCF-7 cells, the ratio of *n*-9/*n*-7 isomers in PC 16:0_16:1 increased after SCD1 inhibition along with increased CAY10566 concentration (Fig. 6B). To provide additional evidence, SCD1 gene silencing was performed, leading to an increased level of *n*-9/*n*-7 isomer ratio in PC 16:0_16:1, consistent with the results observed after CAY10566 treatment (Fig. 6C). Interestingly, MDA-MB-231 cells have a higher proportion of *n*-9 isomers in C16:1-containing GPs compared to the other cell lines (Fig. 6D), suggesting that FAO is more active, which is consistent with previous research [52]. Furthermore, the increases of *n*-9 isomer in C16:1-containing GPs were consistently observed in all human breast cancer cell lines (Fig. 6D). Previous studies showed that the inhibition of SCD1 activates carnitine palmitoyltransferase 1 (CPT1), which is a solute carrier transporter at the outer membrane responsible for transporting FAs into mitochondria to support FAO [53–56], thus leading to increased amounts of C16:1 (*n*-9).

To acquire additional evidence to understand the role of FAO, we analyzed MDA-MB-231 cells, which showed unexpected changes in C18:1 C=C location isomers of GPs upon SCD1 inhibition, to test whether FAO is correlated with *n*-9/*n*-7 composition in C18:1-containing GPs. We inhibited the important rate-limiting enzyme of FAO, CPT1 with etomoxir, and then analyzed C18:1 C=C location isomers of GPs. Results showed that the simultaneous inhibition of CPT1 and SCD1 can successfully increase *n*-9/*n*-7 isomer ratios in FA 18:1 and GPs with C18:1, which is consistent with others when inhibited with SCD1 only (Fig. 6E and Fig. S8). This set of experiments suggests that both lipid desaturation and β-oxidation are involved in the dynamic regulation of C18:1 C=C location isomers.

It is generally believed that triple-negative breast cancer is highly aggressive and the prognosis is worse. Both MDA-MB-468 and MDA-MB-231 cells were triple-negative breast cancer cells from the pleural effusion of women with metastatic breast cancer, but their invasiveness is very different. MDA-MB-231 cells are commonly regarded as highly invasive, as evidenced by the high levels of invasion markers including CXCR4 and stem cell markers (CD44/CD24 and ALDH1^+^), secreted exosomes with high levels of miRNA-122 or 105, interleukin-8 (IL-8), or AKR1B10 [57–62]. However, the levels of these markers were low in MDA-MB-468 cells, which showed a low metastatic potential both in vivo and in vitro [57–60,63]. Therefore, though MDA-MB-468 and MDA-MB-231 cells are both triple-negative, the latter are more flexible in metabolic reprogramming (e.g., FAO) to promote uncontrolled growth and metastasis of cancer cells, and a clear connection of FAO with the metastatic potentials of cancer cells has been established [64–66]. Same as MDA-MB-231 cells that have higher FAO activities revealed by PB-MS/MS, SK-BR-3 cells also have higher metastasis capability, further validating the correlation between FAO and metastasis [60,61,67]. In summary, the in-depth lipidomics analysis has enabled comprehensive analysis of lipids in biological samples at a large scale. Detailed lipid structure characterization is indispensable for accurate lipid pathway mapping, and in this work, we have demonstrated an example of monitoring FAO activity using lipid analysis by quantifying lipid C=C location isomers, which implies information about the invasiveness of cancer cells (Fig. 6F). This workflow is simple, rapid, and straightforward, as it does not require direct protein detection or addition of any internal standard for the relative quantitation of lipid isomers.

## Discussion

SCD1 expression is frequently found to be high in cancer development, accompanied by extensive lipid metabolism reprogramming [9,20,68]. Previous studies have explored the critical role of lipids in the occurrence and development of cancers, including tumor formation, tumor growth, and metastasis [20,69]. From the perspective of lipid unsaturation, saturated FAs were found to trigger endoplasmic reticulum stress response and slow tumor growth [9,70,71], while unsaturated FAs were correlated with cancer presence, poor prognosis, and greater death rates [72,73]. Such findings suggest that both saturated and unsaturated lipids are related to cancer and their specific functions need to be further studied.

The recent progress in lipid analysis tools has importantly improved the coverage of the fine structures that can be resolved through a streamlined experiment workflow. Structural lipidomics is likely to become an essential technique to support the studies of tumor biology, biomarker discovery, and diagnostic applications. It was demonstrated that higher *n*-7/*n*-9 ratios in C18:1-containing lipids were found in colon cancer cell lines, breast cancer tissues, and lung cancer tissue [32,38,47,74,75]. Lower *n*-7/*n*-9 ratios in C18:1-containing lipids were found in the lymph node tissues with thyroid cancer metastasis [39]. Clearly, as shown by this work and others, lipid remodeling, which involves recombination and reconstruction of different carbon chain FAs, is closely correlated with these cancers and a variety of enzymes are involved in lipid metabolism. By comprehensive lipid analysis via PB-MS/MS, we have shown that SCD1 and FAO activities are high in specific subtypes of human breast cancer cell lines, both of which are correlated with cancer metastasis and invasiveness (high metabolic rate). Certainly, the detailed functional outcome of many structurally distinct lipids or a group of remodeled lipids remains to be elucidated, with the emergence of more powerful lipid analysis tools.

In this work, by using LC-PB-MS/MS, we have monitored the lipidome alterations of human breast cancer cells of different subtypes, with key lipid metabolic enzymes including SCD1 inhibited. We were able to reveal how lipid desaturation is involved in regulating the lipidome at the C=C level. By exploring the different responses of different cell lines to SCD1 inhibition, we have demonstrated that lipid β-oxidation also has an important impact on lipid metabolism reprogramming. While the metabolic pathways of lipid metabolism studied here are well-known from a biological point of view, we showed that quantitative lipid C=C location isomer analysis can be conveniently used to probe the activity of key enzymes involved in the metabolism pathways. For instance, the relative amount of *n*-9 isomer of C16:1 is highly correlated with FAO activity and the invasiveness of human breast cancer cells, providing a new perspective for metabolic phenotyping and cancer diagnostics. This study may also contribute to the development of more effective cancer therapies through the inhibition of multiple enzyme targets, e.g., SCD1, FADS2, and CPT1, to minimize cancer recurrence and metastasis.

## Materials and Methods

### Materials and chemicals

Ammonium acetate and dimethyl sulfoxide (DMSO) were purchased from Sigma-Aldrich (St Louis, MO, USA). HCl was purchased from Beijing Chemical Works (Beijing, China). Ammonium hydroxide was purchased from Modern Oriental Fine Chemistry (Beijing, China). All other HPLC-grade organic solvents such as acetone, acetonitrile, water, and formic acid were purchased from Fisher Scientific (NJ, USA) and used without further purification. The CCK-8 assay kit was purchased from BioDee Biotechnology (Beijing, China). DharmaFECT 1 transfection reagent was obtained from Dharmacon Research, Inc. (Cambridge, UK). MCF-7 (catalog number 3111C0001CCC000013) was purchased from the National Infrastructure of Cell Line Resource (Beijing, China). BT-474 (catalog number 3111C0001CCC000129), SK-BR-3 (catalog number 3111C0001CCC000085), MDA-MB-231 (catalog number 3111C0001CCC000014), and MDA-MB-468 (catalog number 3111C0001CCC000249) cells were purchased from Shanghai Enzyme Research Biotechnology Co. Ltd (Shanghai, China). Dulbecco’s modified Eagle’s medium (DMEM), Roswell Park Memorial Institute-1640 medium (RPMI-1640, w/o Hepes), Leibovitz’s L-15 Medium (L-15), DPBS, fetal bovine serum (FBS), and penicillin−streptomycin (100 U·ml^−1^) were purchased from Gibco (Life Technologies, Carlsbad, CA). Small-molecule inhibitors, CAY10566, and etomoxir were purchased from MedChem Express (Monmouth Junction, USA), dissolved in DMSO, and stored at −80 °C.

### Cell culture

MCF-7 and SK-BR-3 cells were cultured in DMEM supplemented with 10% FBS and 1% penicillin–streptomycin. BT-474 cells were cultured in RPMI-1640 medium supplemented with 10% FBS and 1% penicillin–streptomycin. These 3 types of cells were cultured with a breathable dish in a humidified atmosphere containing 5% CO_2_. MDA-MB-468 and MDA-MB-231 cells were cultured in L-15 medium with the same supplements and in sealed culturing dishes. All cells were cultured at 37 °C and passaged every 2 or 3 days. After reaching 90% confluence, cells were detached using 0.1% trypsin solution and collected by centrifugation. Then, 1 ml of methanol was immediately added to quench cellular activity. As for SCD1 and CPT1 inhibition, cells were treated with 0, 10, 100, 300, 500, 800, and 1,000 nM CAY10566, or 30 μM etomoxir in DMSO, while the control group was treated with an equal volume of DMSO.

### Lipid extraction

A modified Folch method was employed for phospholipid extraction from cultured cells. The cell suspension in methanol (1 ml) was sonicated in a water bath for 10 min and then transferred to a 10-ml centrifuge tube diluted with 1 ml of deionized water and 2 ml of chloroform. After 30 s vortex, the mixture was centrifuged at 10,000 *g* for 10 min. The bottom chloroform layer was collected in a borosilicate glass tube. Another 2 ml of chloroform was added to the residual top layer to repeat the extraction process. The bottom layer of chloroform was collected and combined with the previously collected fraction and dried under nitrogen flow. Finally, the dried lipid extract was redissolved with 1 ml of methanol and stored at −20 °C before MS analysis. For free FA extraction, the cell suspension in methanol (1 ml) was sonicated in a water bath for 10 min and then transferred to a 10-ml centrifuge tube. The sample was diluted with 300 μl of deionized water and acidified with HCl to 25 mM final concentration. After adding 1 ml of isooctane, the mixture was vortexed and centrifuged at 3,000 *g* for 1 min. The top layer was transferred to a glass tube and dried under nitrogen flow. Finally, extracted free FAs were redissolved with 100 μl of methanol and stored at −20 °C before MS analysis.

### Lipidomics analysis

The analysis of phospholipids was performed on an LC–PB–MS/MS system, including a 4500 QTRAP triple quadrupole/linear ion trap hybrid mass spectrometer (Applied Biosystems/Sciex, Toronto, Canada), an ExionLC AC system (Sciex, Toronto, CA), and a home-built flow microreactor. The flow microreactor used for post-column online PB derivation is made from fluorinated ethylene propylene tubing (0.03-in. internal diameter, 1/16 outer diameter) and a low-pressure mercury lamp with emission centered around 254 nm (Model No.: 80-1057-01, BHK, Inc., CA, USA). Lipid separation was performed on a hydrophilic interaction chromatography (HILIC) column (150 mm × 2.1 mm, silica spheres, 2.7 μm) from Sigma-Aldrich (St. Louis, MO, USA). The column temperature was set at 30 °C. The mobile phase consisted of (A) acetone/ACN/HAc (50/50/0.2, v/v/v) and (B) ammonium acetate aqueous solution (10 mM). Gradient elution was applied for separation (A started from 90%, decreased to 85% at 5 min, then decreased to 80% at 8 min, kept at 80% within 8 to 15 min, and decreased to 70% in 16 min and kept this percentage to 20 min) at a flow rate of 0.2 ml min^−1^. For MS analysis, each sample was analyzed by 3 steps. Based on the first step, LC-MS/MS, i.e., neutral loss scan (NLS) 141 for PEs, NLS 185 for PSs, and precursor ion scan (PIS) 184 for PCs, a list of potential PB precursors was provided to guide the second online LC-PB-MS/MS step. For *n*-10 isomer validation in BT-474 cells, LC-PB-MS/MS/MS was applied. The third step was GP chain information analysis, which was conducted by LC-MS/MS in negative ion mode. For the preliminary screen of C16:1- and C18:1-containing GPs, PIS or NLS was applied in negative ion mode or positive ion mode in the presence of 1 mM LiOH, which was added to eliminate counterparts. The analysis of free FA was conducted by online PB reaction coupled with nano-electrospray ionization (nanoESI). The nanoESI tip was aligned with the sampling orifice of the mass spectrometer. To initiate PB reaction, a low-pressure mercury lamp was set 1.0 cm over the tip. Before PB reaction, the FA extract was dried and redissolved in acetone:water (7:3, v/v) added with 0.1% ammonium hydroxide.

### siRNA-mediated gene knockdown strategy

MCF-7 cells were suspended in DMEM without FBS and then transfected using Sangon Biotech siRNA (75nM final concentration) via DharmaFECT 1 transfection reagent using the manufacturer’s protocol in 6-well format. After 24 h, transfected cells were cultured in a complete DMEM for another 24 h and collected for analysis. Knockdown efficiency was approximately 65% as measured by quantitative real-time polymerase chain reaction (PCR) analysis after 48 h transfection.

### CCK-8 assay

The cytotoxicity of CAY10566 was evaluated by CCK-8 assay. Briefly, MCF-7 cells were seeded into 96-well plates at a density of 2,000 cells/well and a volume of 100 μl. Next, 10 μl per well of CCK-8 solution was added. Then, the cells were cultured for another 2 h at 37 °C and the absorbance at 450 nm was recorded according to the manufacturer’s instructions.

### Data analysis

For the relative quantitative analysis of GPs C=C location isomers, the peak areas of corresponding diagnostic ions were used. Hierarchical cluster analysis was conducted using HemI67 software (version 1.0.3.7) [76], with Pearson's correlation coefficient for distance measurements and average linkage (default) variance in the clustering method.

## Data Availability

All data used to support the findings of this study are available from the corresponding author upon request.

## Supplementary Materials

Supplementary 1 Fig. S1. Mass spectra of the PE profile in MCF-7 cells resulting from *m*/*z* 141 NLS before and after SCD1 inhibition. Fig. S2. (A) Cell density and morphology of MCF-7 cells before and after SCD1 inhibition. Fig. S3. Lipid C=C analysis of BT-474 cells via LC-PB-MS/MS/MS in negative ion mode. Fig. S4. The relative expression levels of both FADS2 and SCD1 analyzed by the CCLE database. Fig. S5. Compositional changes of C=C location isomers in C18:1-containing PEs (A) and PSs (B) after siRNA treatment of SCD1. Fig. S6. Changes in GPs chain composition in MCF-7 cells analyzed by LC-MS/MS in negative ion mode. Fig. S7. A 2-dimensional electrospray ionization mass spectrometric approach to screen C16:1 GPs with the minimum influence of C18:1 GPs, in the presence of 1 mM LiOH. Fig. S8. CPT1 inhibition by etomoxir rescues the decrease of FA18:1 *n*-9/*n*-7 isomers due to SCD1 inhibition in MDA-MB-231 cells. Table S1. C18:1-containing GPs with C=C location isomers in MCF-7cells. Table S2. C18:1-containing GPs with C=C location isomers in MDA-MB-468 cells. Table S3. C18:1-containing GPs with C=C location isomers in BT-474 cells. Table S4. C18:1-containing GPs with C=C location isomers in SK-BR-3 cells. Table S5. C18:1-containing GPs with C=C location isomers in MDA-MB-231 cells.
