# Supplementary material for: Metabolic Pathway of Monounsaturated Lipids Revealed by In-Depth Structural Lipidomics by Mass Spectrometry
Source: Research (Wash D C). 2023 Mar 15;6:0087. doi: 10.34133/research.0087 (PMC10026824; doi:10.34133/research.0087)
Supplement: Supplementary 1 — Fig. S1. Mass spectra of the PE profile in MCF-7 cells resulting from m/z 141 NLS before and after SCD1 inhibition. Fig. S2. (A) Cell density and morphology of MCF-7 cells before and after SCD1 inhibition. Fig. S3. Lipid C=C analysis of BT-474 cells via LC-PB-MS/MS/MS in negative ion mode. Fig. S4. The relative expression levels of both FADS2 and SCD1 analyzed by the CCLE database. Fig. S5. Compositional changes of C=C location isomers in C18:1-containing PEs (A) and PSs (B) after siRNA treatment of SCD1. Fig. S6. Changes in GPs chain composition in MCF-7 cells analyzed by LC-MS/MS in negative ion mode. Fig. S7. A 2-dimensional electrospray ionization mass spectrometric approach to screen C16:1 GPs with the minimum influence of C18:1 GPs, in the presence of 1 mM LiOH. Fig. S8. CPT1 inhibition by etomoxir rescues the decrease of FA18:1 n-9/n-7 isomers due to SCD1 inhibition in MDA-MB-231 cells. Table S1. C18:1-containing GPs with C=C location isomers in MCF-7cells. Table S2. C18:1-containing GPs with C=C location isomers in MDA-MB-468 cells. Table S3. C18:1-containing GPs with C=C location isomers in BT-474 cells. Table S4. C18:1-containing GPs with C=C location isomers in SK-BR-3 cells. Table S5. C18:1-containing GPs with C=C location isomers in MDA-MB-231 cells. [file research.0087.f1.docx]

**Supporting information for**

Lipid Metabolic Pathway Study Assisted by In-depth Structural Lipidomics

Simin Cheng^1^, Donghui Zhang^1^, Jiaxin Feng^2^, Qingyuan Hu^1^, Aolei Tan^1^, Zhuoning Xie^1^, Qinhua Chen^3^, Huimin Huang^4^, Ying Wei^4^, Zheng Ouyang^1^*, Xiaoxiao Ma^1^*

^1^State Key Laboratory of Precision Measurement Technology and Instruments, Department of Precision Instrument, Tsinghua University, Beijing, 100084, China.

^2^Department of Chemistry, Tsinghua University, Beijing, 100084, China.

^3^Key Laboratory of TCM Clinical Pharmacy, Shenzhen Baoan Authentic TCM Therapy Hospital, Shenzhen, Guangdong, 518101, China

^4^Sinopharm Dongfeng General Hospital, Hubei University of Medicine, Experiment center of medicine, Shiyan, Hubei, 442008, China

Correspondence should be addressed to Zheng Ouyang; ouyang@tsinghua.edu.cn and Xiaoxiao Ma; maxx@tsinghua.edu.c

Fig. S1. Mass spectra of PE profile in MCF-7 cells resulting from m/z 141 NLS before and after SCD1 inhibition.

Fig. S2. (a) Cell density and morphology of MCF-7 cells before and after SCD1 inhibition. White arrows: pseudopodia. (b) MCF-7 cell viability analysis by CCK-8 assay. No detailed measurements were performed for 800 nM and 1000 nM, because of the slow cell growth rate and low cell density.

**Fig. S3.** Lipid C=C analysis of BT-474 cells via LC-PB-MS/MS/MS in negative ion mode. (a-c) LC-PB-MS/MS/MS spectra of PC 34:1 and the corresponding structures before and after SCD1 inhibition in BT-474 cells. Compositional changes of C=C location isomers among PC 16:1_16:1 (d), PC 16:0_16:1 (e), PC 16:1_18:0 (f), PC 16:0_18:1 (g), PC 18:1_18:1 (h), PC 18:0_18:1 (i), PE 16:0_16:1 (j), PE 14:0_18:1 (k), PE 16:1_18:0 (l), PE 16:0_18:1 (m), PE 18:1_18:1 (n) and PE 18:0_18:1 (o).

**Fig. S4.** The relative expression levels of both FADS2 and SCD1 analyzed by CCLE database. TPM: Transcripts Per Kilobase of exon model per Million mapped reads.

**Fig. S5.** Compositional changes of C=C location isomers in C18:1-containing PEs (a) and PSs (b) after siRNA treatment of SCD1. (c) RT-PCR measurement of SCD1 mRNA level in MCF-7 cells transfected with SCD1 siRNA.

**Fig. S6.** Changes in GPs chain composition in MCF-7 cells analyzed by LC-MS/MS in negative ion mode. (a) LC-MS/MS spectra of PC 36:3 in negative ion mode under series of CAY10566 concentrations. (b) Compositional changes of chain composition isomers before and after SCD1 inhibition under different CAY10566 concentrations.

**Fig. S7.** A two-dimensional electrospray ionization mass spectrometric approach to screen C16:1 GPs with the minimum influence of C18:1 GPs, in the presence of 1 mM LiOH. (a) NLS of m/z 254 in positive ion mode to screen PCs with fatty acyl C16:1. (b) PIS of m/z 253 in negative ion mode to screen PEs with fatty acyl C16:1. (c) NLS of m/z 282 in positive ion mode to screen PCs with fatty acyl C18:1. (d) PIS of m/z 281 in negative ion mode to screen PEs with fatty acyl C18:1.

**Fig. S8.** CPT1 inhibition by etomoxir rescues the decrease of FA18:1 n-9/n-7 isomers due to SCD1 inhibition in MDA-MB-231 cells.

Table S1. C18:1-containing GPs with C=C location isomers in MCF-7cells.

| **Lipid**  **Subclass** | **[M+H]^+^ *m/z*** | **PB precursor *m/z*** | **Diagnostic ions**  ***m/z*** | **C=C location Isomers** |
| --- | --- | --- | --- | --- |
| PC | 732.5 | 790.5 | 608, 634 | PC 16:0_16:1(n-10) |
|  |  |  | 622, 648 | PC 16:0_16:1(n-9) |
|  |  |  | 650, 676 | PC 16:0_16:1(n-7) |
|  | 746.5 | 804.5 | 636, 662 | PC 15:0_18:1(n-9) |
|  |  |  | 664, 690 | PC 15:0_18:1(n-7) |
|  | 760.5 | 818.5 | 636, 662 | PC 16:0_18:1(n-10) |
|  |  |  | 650, 676 | PC 16:0_18:1(n-9) |
|  |  |  | 678, 704 | PC 16:0_18:1(n-7) |
|  | 772.5 | 830.5 | 662, 688 | PC 17:1_18:1(n-9) |
|  |  |  | 690, 716 | PC 17:1_18:1(n-7) |
|  | 774.5 | 832.5 | 664, 690 | PC 17:0_18:1(n-9) |
|  |  |  | 692, 718 | PC 17:0_18:1(n-7) |
|  | 784.5 | 842.5 | 674, 700 | PC 18:1(n-9)_18:2 |
|  |  |  | 702, 728 | PC 18:1(n-7)_18:2 |
|  | 786.5 | 844.5 | 676, 702 | PC 18:1(n-9)_18:1(n-9) |
|  |  |  | 704, 730 | PC 18:1(n-7)_18:1(n-7) |
|  | 788.5 | 846.5 | 664, 690 | PC 18:0_18:1(n-10) |
|  |  |  | 678, 704 | PC 18:0_18:1(n-9) |
|  |  |  | 706, 732 | PC 18:0_18:1(n-7) |
|  | 800.5 | 858.5 | 690, 716 | PC 18:1(n-9)_19:1 |
|  |  |  | 718, 744 | PC 18:1(n-7)_19:1 |
|  | 802.5 | 860.5 | 692, 718 | PC 18:1(n-9)_19:0 |
|  |  |  | 720, 746 | PC 18:1(n-7)_19:0 |
|  | 814.5 | 872.5 | 704, 730 | PC 18:1(n-9)_20:1 |
|  |  |  | 732, 758 | PC 18:1(n-7)_20:1 |
|  | 816.5 | 874.5 | 706, 732 | PC 18:1(n-9)_20:0 |
|  |  |  | 734, 760 | PC 18:1(n-7)_20:0 |
| PE | 704.5 | 762.5 | 453, 479 | PE 15:0_18:1(n-9) |
|  |  |  | 481, 507 | PE 15:0_18:1(n-7) |
|  | 716.5 | 774.5 | 465, 491 | PE 16:1_18:1(n-9) |
|  |  |  | 493, 519 | PE 16:1_18:1(n-7) |
|  | 718.5 | 776.5 | 467, 493 | PE 16:0_18:1(n-9) |
|  |  |  | 495, 521 | PE 16:0_18:1(n-7) |
|  | 730.5 | 788.5 | 479, 505 | PE 17:1_18:1(n-9) |
|  |  |  | 507, 533 | PE 17:1_18:1(n-7) |
|  | 732.5 | 790.5 | 481, 507 | PE 17:0_18:1(n-9) |
|  |  |  | 509, 535 | PE 17:0_18:1(n-7) |
|  | 742.5 | 800.5 | 491, 517 | PE 18:1(n-9)_18:2 |
|  |  |  | 519, 545 | PE 18:1(n-7)_18:2 |
|  | 744.5 | 802.5 | 493, 519 | PE 18:1(n-9)_18:1(n-9) |
|  |  |  | 521, 547 | PE 18:1(n-7)_18:1(n-7) |
|  | 746.5 | 804.5 | 481, 507 | PE 18:0_18:1(n-10) |
|  |  |  | 495, 521 | PE 18:0_18:1(n-9) |
|  |  |  | 523, 549 | PE 18:0_18:1(n-7) |
|  | 758.5 | 816.5 | 507, 533 | PE 18:1(n-9)_19:1 |
|  |  |  | 535, 561 | PE 18:1(n-7)_19:1 |
|  | 760.6 | 818.5 | 509, 535 | PE 18:1(n-9)_19:0 |
|  |  |  | 537, 563 | PE 18:1(n-7)_19:0 |
|  | 770.5 | 828.5 | 519, 545 | PE 18:1(n-9)_20:2 |
|  |  |  | 547, 573 | PE 18:1(n-7)_20:2 |
|  | 772.5 | 830.5 | 521, 547 | PE 18:1(n-9)_20:1 |
|  |  |  | 549, 575 | PE 18:1(n-7)_20:1 |
|  | 774.5 | 832.5 | 523, 549 | PE 18:1(n-9)_20:0 |
|  |  |  | 551, 577 | PE 18:1(n-7)_20:0 |
| PS | 762.5 | 820.5 | 467, 493 | PS 16:0_18:1(n-9) |
|  |  |  | 495, 521 | PS 16:0_18:1(n-7) |
|  | 788.5 | 846.5 | 493, 519 | PS 18:1(n-9)_18:1(n-9) |
|  |  |  | 521, 547 | PS 18:1(n-7)_18:1(n-7) |
|  | 790.5 | 848.5 | 495, 521 | PS 18:0_18:1(n-9) |
|  |  |  | 523, 549 | PS 18:0_18:1(n-7) |
|  | 816.5 | 874.5 | 521, 547 | PS 18:1(n-9)_20:1 |
|  |  |  | 549, 575 | PS 18:1(n-7)_20:1 |
|  | 844.5 | 902.5 | 549, 575 | PS 18:1(n-9)_22:1 |
|  |  |  | 577, 603 | PS 18:1(n-7)_22:1 |
| **Lipid**  **Subclass** | **[M-H]^-^ *m/z*** | **PB precursor *m/z*** | **Diagnostic ions**  ***m/z*** | **C=C location Isomers** |
| FA | 281.2 | 339.2 | 179, 197 | FA 18:1(n-9) |
|  |  |  | 199, 225 | FA 18:1(n-7) |

Table S2. C18:1-containing GPs with C=C location isomers in MDA-MB-468 cells.

| **Lipid**  **Subclass** | **[M+H]^+^ *m/z*** | ***PB precursor m/z*** | **Diagnostic ions**  ***m/z*** | **C=C location Isomers** |
| --- | --- | --- | --- | --- |
| PC | 732.5 | 790.5 | 608, 634 | PC 16:0_16:1(n-10) |
|  |  |  | 622, 648 | PC 16:0_16:1(n-9) |
|  |  |  | 650, 676 | PC 16:0_16:1(n-7) |
|  | 746.5 | 804.5 | 636, 662 | PC 15:0_18:1(n-9) |
|  |  |  | 664, 690 | PC 15:0_18:1(n-7) |
|  | 758.5 | 816.5 | 648, 674 | PC 16:1_18:1(n-9) |
|  |  |  | 676, 702 | PC 16:1_18:1(n-7) |
|  | 760.5 | 818.5 | 636, 662 | PC 16:0_18:1(n-10) |
|  |  |  | 650, 676 | PC 16:0_18:1(n-9) |
|  |  |  | 678, 704 | PC 16:0_18:1(n-7) |
|  | 772.5 | 830.5 | 662, 688 | PC 17:1_18:1(n-9) |
|  |  |  | 690, 716 | PC 17:1_18:1(n-7) |
|  | 774.5 | 832.5 | 664, 690 | PC 17:0_18:1(n-9) |
|  |  |  | 692, 718 | PC 17:0_18:1(n-7) |
|  | 784.5 | 842.5 | 674, 700 | PC 18:1(n-9)_18:2 |
|  |  |  | 702, 728 | PC 18:1(n-7)_18:2 |
|  | 786.5 | 844.5 | 676, 702 | PC 18:1(n-9)_18:1(n-9) |
|  |  |  | 704, 730 | PC 18:1(n-7)_18:1(n-7) |
|  | 788.5 | 846.5 | 664, 690 | PC 18:0_18:1(n-10) |
|  |  |  | 678, 704 | PC 18:0_18:1(n-9) |
|  |  |  | 706, 732 | PC 18:0_18:1(n-7) |
|  | 800.5 | 858.5 | 690, 716 | PC 18:1(n-9)_19:1 |
|  |  |  | 718, 744 | PC 18:1(n-7)_19:1 |
|  | 802.5 | 860.5 | 692, 718 | PC 18:1(n-9)_19:0 |
|  |  |  | 720, 746 | PC 18:1(n-7)_19:0 |
|  | 812.5 | 870.5 | 702, 728 | PC 18:1(n-9)_20:2 |
|  |  |  | 730, 756 | PC 18:1(n-7)_20:2 |
|  | 814.5 | 872.5 | 704, 730 | PC 18:1(n-9)_20:1 |
|  |  |  | 732, 758 | PC 18:1(n-7)_20:1 |
|  | 816.5 | 874.5 | 706, 732 | PC 18:1(n-9)_20:0 |
|  |  |  | 734, 760 | PC 18:1(n-7)_20:0 |
| PE | 704.6 | 762.6 | 453, 479 | PE 15:0_18:1(n-9) |
|  |  |  | 481, 507 | PE 15:0_18:1(n-7) |
|  | 716.5 | 774.5 | 465, 491 | PE 16:1_18:1(n-9) |
|  |  |  | 493, 519 | PE 16:1_18:1(n-7) |
|  | 718.5 | 776.5 | 467, 493 | PE 16:0_18:1(n-9) |
|  |  |  | 495, 521 | PE 16:0_18:1(n-7) |
|  | 730.5 | 788.5 | 479, 505 | PE 17:1_18:1(n-9) |
|  |  |  | 507, 533 | PE 17:1_18:1(n-7) |
|  | 732.5 | 790.5 | 481, 507 | PE 17:0_18:1(n-9) |
|  |  |  | 509, 535 | PE 17:0_18:1(n-7) |
|  | 742.5 | 800.5 | 491, 517 | PE 18:1(n-9)_18:2 |
|  |  |  | 519, 545 | PE 18:1(n-7)_18:2 |
|  | 744.5 | 802.5 | 493, 519 | PE 18:1(n-9)_18:1(n-9) |
|  |  |  | 521, 547 | PE 18:1(n-7)_18:1(n-7) |
|  | 746.5 | 804.5 | 481, 507 | PE 18:0_18:1(n-10) |
|  |  |  | 495, 521 | PE 18:0_18:1(n-9) |
|  |  |  | 523, 549 | PE 18:0_18:1(n-7) |
|  | 758.5 | 816.5 | 507, 533 | PE 18:1(n-9)_19:1 |
|  |  |  | 535, 561 | PE 18:1(n-7)_19:1 |
|  | 760.6 | 818.5 | 509, 535 | PE 18:1(n-9)_19:0 |
|  |  |  | 537, 563 | PE 18:1(n-7)_19:0 |
|  | 770.5 | 828.5 | 519, 545 | PE 18:1(n-9)_20:2 |
|  |  |  | 547, 573 | PE 18:1(n-7)_20:2 |
|  | 772.5 | 830.5 | 521, 547 | PE 18:1(n-9)_20:1 |
|  |  |  | 549, 575 | PE 18:1(n-7)_20:1 |
| PS | 762.5 | 820.5 | 467, 493 | PS 16:0_18:1(n-9) |
|  |  |  | 495, 521 | PS 16:0_18:1(n-7) |
|  | 788.5 | 846.5 | 493, 519 | PS 18:1(n-9)_18:1(n-9) |
|  |  |  | 521, 547 | PS 18:1(n-7)_18:1(n-7) |
|  | 790.5 | 848.5 | 495, 521 | PS 18:0_18:1(n-9) |
|  |  |  | 523, 549 | PS 18:0_18:1(n-7) |
| **Lipid**  **Subclass** | **[M-H]^-^ *m/z*** | **PB precursor *m/z*** | **Diagnostic ions**  ***m/z*** | **C=C location Isomers** |
| FA | 281.2 | 339.2 | 179, 197 | FA 18:1(n-9) |
|  |  |  | 199, 225 | FA 18:1(n-7) |

Table S3. C18:1-containing GPs with C=C location isomers in BT-474 cells.

| **Lipid**  **Subclass** | **[M+H]^+^ *m/z*** | **PB precursor *m/z*** | **Diagnostic ions**  ***m/z*** | **C=C location Isomers** |
| --- | --- | --- | --- | --- |
| PC | 730.5 | 788.5 | 606, 632 | PC 16:1(n-10)_16:1(n-10) |
|  |  |  | 620, 646 | PC 16:1(n-9)_16:1(n-9) |
|  |  |  | 648, 674 | PC 16:1(n-7)_16:1(n-7) |
|  | 732.5 | 790.5 | 608, 634 | PC 16:0_16:1(n-10) |
|  |  |  | 622, 648 | PC 16:0_16:1(n-9) |
|  |  |  | 650, 676 | PC 16:0_16:1(n-7) |
|  | 758.5 | 816.5 | 648, 674 | PC 16:1_18:1(n-9) |
|  |  |  | 676, 702 | PC 16:1_18:1(n-7) |
|  | 760.5 | 818.5 | 636, 662 | PC 16:0_18:1(n-10) |
|  |  |  | 650, 676 | PC 16:0_18:1(n-9) |
|  |  |  | 678, 704 | PC 16:0_18:1(n-7) |
|  | 774.5 | 832.5 | 664, 690 | PC 17:0_18:1(n-9) |
|  |  |  | 692, 718 | PC 17:0_18:1(n-7) |
|  | 784.5 | 842.5 | 674, 700 | PC 18:1(n-9)_18:2 |
|  |  |  | 702, 728 | PC 18:1(n-7)_18:2 |
|  | 786.5 | 844.5 | 662, 688 | PC 18:1(n-10)_18:1(n-10) |
|  |  |  | 676, 702 | PC 18:1(n-9)_18:1(n-9) |
|  |  |  | 704, 730 | PC 18:1(n-7)_18:1(n-7) |
|  | 788.5 | 846.5 | 664, 690 | PC 18:0_18:1(n-10) |
|  |  |  | 678, 704 | PC 18:0_18:1(n-9) |
|  |  |  | 706, 732 | PC 18:0_18:1(n-7) |
|  | 800.5 | 858.5 | 690, 716 | PC 18:1(n-9)_19:1 |
|  |  |  | 718, 744 | PC 18:1(n-7)_19:1 |
|  | 802.5 | 860.5 | 692, 718 | PC 18:1(n-9)_19:0 |
|  |  |  | 720, 746 | PC 18:1(n-7)_19:0 |
|  | 812.5 | 870.5 | 702, 728 | PC 18:1(n-9)_20:2 |
|  |  |  | 730, 756 | PC 18:1(n-7)_20:2 |
|  | 814.5 | 872.5 | 704, 730 | PC 18:1(n-9)_20:1 |
|  |  |  | 732, 758 | PC 18:1(n-7)_20:1 |
| PE | 690.5 | 748.5 | 425, 451 | PE 16:0_16:1(n-10) |
|  | 704.6 | 762.6 | 453, 479 | PE 15:0_18:1(n-9) |
|  |  |  | 481, 507 | PE 15:0_18:1(n-7) |
|  | 716.5 | 774.5 | 465, 491 | PE 16:1_18:1(n-9) |
|  |  |  | 493, 519 | PE 16:1_18:1(n-7) |
|  | 718.5 | 776.5 | 467, 493 | PE 16:0_18:1(n-9) |
|  |  |  | 495, 521 | PE 16:0_18:1(n-7) |
|  | 730.5 | 788.5 | 479, 505 | PE 17:1_18:1(n-9) |
|  |  |  | 507, 533 | PE 17:1_18:1(n-7) |
|  | 732.5 | 790.5 | 481, 507 | PE 17:0_18:1(n-9) |
|  |  |  | 509, 535 | PE 17:0_18:1(n-7) |
|  | 742.5 | 800.5 | 491, 517 | PE 18:1(n-9)_18:2 |
|  |  |  | 519, 545 | PE 18:1(n-7)_18:2 |
|  | 744.5 | 802.5 | 479, 505 | PE 18:1(n-10)_18:1(n-10) |
|  |  |  | 493, 519 | PE 18:1(n-9)_18:1(n-9) |
|  |  |  | 521, 547 | PE 18:1(n-7)_18:1(n-7) |
|  | 746.5 | 804.5 | 481, 507 | PE 18:0_18:1(n-10) |
|  |  |  | 495, 521 | PE 18:0_18:1(n-9) |
|  |  |  | 523, 549 | PE 18:0_18:1(n-7) |
|  | 758.5 | 816.5 | 507, 533 | PE 18:1(n-9)_19:1 |
|  |  |  | 535, 561 | PE 18:1(n-7)_19:1 |
|  | 760.6 | 818.5 | 509, 535 | PE 18:1(n-9)_19:0 |
|  |  |  | 537, 563 | PE 18:1(n-7)_19:0 |
|  | 772.5 | 830.5 | 521, 547 | PE 18:1(n-9)_20:1 |
|  |  |  | 549, 575 | PE 18:1(n-7)_20:1 |
| PS | 762.5 | 820.5 | 467, 493 | PS 16:0_18:1(n-9) |
|  |  |  | 495, 521 | PS 16:0_18:1(n-7) |
|  | 788.5 | 846.5 | 493, 519 | PS 18:1(n-9)_18:1(n-9) |
|  |  |  | 521, 547 | PS 18:1(n-7)_18:1(n-7) |
|  | 790.5 | 848.5 | 481, 507 | PS 18:0_18:1(n-10) |
|  |  |  | 495, 521 | PS 18:0_18:1(n-9) |
|  |  |  | 523, 549 | PS 18:0_18:1(n-7) |
| **Lipid**  **Subclass** | **[M-H]^-^ *m/z*** | **PB precursor *m/z*** | **Diagnostic ions**  ***m/z*** | **C=C location Isomers** |
| FA | 281.2 | 339.2 | 179, 197 | FA 18:1(n-9) |
|  |  |  | 199, 225 | FA 18:1(n-7) |

Table S4. C18:1-containing GPs with C=C location isomers in SK-BR-3 cells.

| **Lipid**  **Subclass** | **[M+H]^+^ *m/z*** | **PB precursor *m/z*** | **Diagnostic ions**  ***m/z*** | **C=C location Isomers** |
| --- | --- | --- | --- | --- |
| PC | 732.5 | 790.5 | 608, 634 | PC 16:0_16:1(n-10) |
|  |  |  | 622, 648 | PC 16:0_16:1(n-9) |
|  |  |  | 650, 676 | PC 16:0_16:1(n-7) |
|  | 746.5 | 804.5 | 636, 662 | PC 15:0_18:1(n-9) |
|  |  |  | 664, 690 | PC 15:0_18:1(n-7) |
|  | 758.5 | 816.5 | 648, 674 | PC 16:1_18:1(n-9) |
|  |  |  | 676, 702 | PC 16:1_18:1(n-7) |
|  | 760.5 | 818.5 | 636, 662 | PC 16:0_18:1(n-10) |
|  |  |  | 650, 676 | PC 16:0_18:1(n-9) |
|  |  |  | 678, 704 | PC 16:0_18:1(n-7) |
|  | 772.5 | 830.5 | 662, 688 | PC 17:1_18:1(n-9) |
|  |  |  | 690, 716 | PC 17:1_18:1(n-7) |
|  | 774.5 | 832.5 | 664, 690 | PC 17:0_18:1(n-9) |
|  |  |  | 692, 718 | PC 17:0_18:1(n-7) |
|  | 784.5 | 842.5 | 674, 700 | PC 18:1(n-9)_18:2 |
|  |  |  | 702, 728 | PC 18:1(n-7)_18:2 |
|  | 786.5 | 844.5 | 676, 702 | PC 18:1(n-9)_18:1(n-9) |
|  |  |  | 704, 730 | PC 18:1(n-7)_18:1(n-7) |
|  | 788.5 | 846.5 | 664, 690 | PC 18:0_18:1(n-10) |
|  |  |  | 678, 704 | PC 18:0_18:1(n-9) |
|  |  |  | 706, 732 | PC 18:0_18:1(n-7) |
|  | 800.5 | 858.5 | 690, 716 | PC 18:1(n-9)_19:1 |
|  |  |  | 718, 744 | PC 18:1(n-7)_19:1 |
|  | 802.5 | 860.5 | 692, 718 | PC 18:1(n-9)_19:0 |
|  |  |  | 720, 746 | PC 18:1(n-7)_19:0 |
| PE | 704.6 | 762.6 | 453, 479 | PE 15:0_18:1(n-9) |
|  |  |  | 481, 507 | PE 15:0_18:1(n-7) |
|  | 716.5 | 774.5 | 465, 491 | PE 16:1_18:1(n-9) |
|  |  |  | 493, 519 | PE 16:1_18:1(n-7) |
|  | 718.5 | 776.5 | 467, 493 | PE 16:0_18:1(n-9) |
|  |  |  | 495, 521 | PE 16:0_18:1(n-7) |
|  | 732.5 | 790.5 | 481, 507 | PE 17:0_18:1(n-9) |
|  |  |  | 509, 535 | PE 17:0_18:1(n-7) |
|  | 742.5 | 800.5 | 491, 517 | PE 18:1(n-9)_18:2 |
|  |  |  | 519, 545 | PE 18:1(n-7)_18:2 |
|  | 744.5 | 802.5 | 493, 519 | PE 18:1(n-9)_18:1(n-9) |
|  |  |  | 521, 547 | PE 18:1(n-7)_18:1(n-7) |
|  | 746.5 | 804.5 | 481, 507 | PE 18:0_18:1(n-10) |
|  |  |  | 495, 521 | PE 18:0_18:1(n-9) |
|  |  |  | 523, 549 | PE 18:0_18:1(n-7) |
|  | 758.5 | 816.5 | 507, 533 | PE 18:1(n-9)_19:1 |
|  |  |  | 535, 561 | PE 18:1(n-7)_19:1 |
|  | 760.6 | 818.5 | 509, 535 | PE 18:1(n-9)_19:0 |
|  |  |  | 537, 563 | PE 18:1(n-7)_19:0 |
|  | 770.5 | 828.5 | 519, 545 | PE 18:1(n-9)_20:2 |
|  |  |  | 547, 573 | PE 18:1(n-7)_20:2 |
|  | 772.5 | 830.5 | 521, 547 | PE 18:1(n-9)_20:1 |
|  |  |  | 549, 575 | PE 18:1(n-7)_20:1 |
| PS | 762.5 | 820.5 | 467, 493 | PS 16:0_18:1(n-9) |
|  |  |  | 495, 521 | PS 16:0_18:1(n-7) |
|  | 788.5 | 846.5 | 493, 519 | PS 18:1(n-9)_18:1(n-9) |
|  |  |  | 521, 547 | PS 18:1(n-7)_18:1(n-7) |
|  | 790.5 | 848.5 | 495, 521 | PS 18:0_18:1(n-9) |
|  |  |  | 523, 549 | PS 18:0_18:1(n-7) |
| **Lipid**  **Subclass** | **[M-H]^-^ *m/z*** | **PB precursor *m/z*** | **Diagnostic ions**  ***m/z*** | **C=C location Isomers** |
| FA | 281.2 | 339.2 | 179, 197 | FA 18:1(n-9) |
|  |  |  | 199, 225 | FA 18:1(n-7) |

Table S5. C18:1-containing GPs with C=C location isomers in MDA-MB-231 cells.

| **Lipid**  **Subclass** | **[M+H]^+^ *m/z*** | **PB precursor *m/z*** | **Diagnostic ions**  ***m/z*** | **C=C location Isomers** |
| --- | --- | --- | --- | --- |
| PC | 732.5 | 790.5 | 608, 634 | PC 16:0_16:1(n-10) |
|  |  |  | 622, 648 | PC 16:0_16:1(n-9) |
|  |  |  | 650, 676 | PC 16:0_16:1(n-7) |
|  | 746.5 | 804.5 | 636, 662 | PC 15:0_18:1(n-9) |
|  |  |  | 664, 690 | PC 15:0_18:1(n-7) |
|  | 758.5 | 816.5 | 648, 674 | PC 16:1_18:1(n-9) |
|  |  |  | 676, 702 | PC 16:1_18:1(n-7) |
|  | 760.5 | 818.5 | 636, 662 | PC 16:0_18:1(n-10) |
|  |  |  | 650, 676 | PC 16:0_18:1(n-9) |
|  |  |  | 678, 704 | PC 16:0_18:1(n-7) |
|  | 772.5 | 830.5 | 662, 688 | PC 17:1_18:1(n-9) |
|  |  |  | 690, 716 | PC 17:1_18:1(n-7) |
|  | 774.5 | 832.5 | 664, 690 | PC 17:0_18:1(n-9) |
|  |  |  | 692, 718 | PC 17:0_18:1(n-7) |
|  | 784.5 | 842.5 | 674, 700 | PC 18:1(n-9)_18:2 |
|  |  |  | 702, 728 | PC 18:1(n-7)_18:2 |
|  | 786.5 | 844.5 | 676, 702 | PC 18:1(n-9)_18:1(n-9) |
|  |  |  | 704, 730 | PC 18:1(n-7)_18:1(n-7) |
|  | 788.5 | 846.5 | 664, 690 | PC 18:0_18:1(n-10) |
|  |  |  | 678, 704 | PC 18:0_18:1(n-9) |
|  |  |  | 706, 732 | PC 18:0_18:1(n-7) |
|  | 800.5 | 858.5 | 690, 716 | PC 18:1(n-9)_19:1 |
|  |  |  | 718, 744 | PC 18:1(n-7)_19:1 |
|  | 802.5 | 860.5 | 692, 718 | PC 18:1(n-9)_19:0 |
|  |  |  | 720, 746 | PC 18:1(n-7)_19:0 |
|  | 812.5 | 870.5 | 702, 728 | PC 18:1(n-9)_20:2 |
|  |  |  | 730, 756 | PC 18:1(n-7)_20:2 |
|  | 814.5 | 872.5 | 704, 730 | PC 18:1(n-9)_20:1 |
|  |  |  | 732, 758 | PC 18:1(n-7)_20:1 |
|  | 816.5 | 874.5 | 706, 732 | PC 18:1(n-9)_20:0 |
|  |  |  | 734, 760 | PC 18:1(n-7)_20:0 |
| PE | 716.5 | 774.5 | 465, 491 | PE 16:1_18:1(n-9) |
|  |  |  | 493, 519 | PE 16:1_18:1(n-7) |
|  | 718.5 | 776.5 | 467, 493 | PE 16:0_18:1(n-9) |
|  |  |  | 495, 521 | PE 16:0_18:1(n-7) |
|  | 742.5 | 800.5 | 491, 517 | PE 18:1(n-9)_18:2 |
|  |  |  | 519, 545 | PE 18:1(n-7)_18:2 |
|  | 744.5 | 802.5 | 493, 519 | PE 18:1(n-9)_18:1(n-9) |
|  |  |  | 521, 547 | PE 18:1(n-7)_18:1(n-7) |
|  | 746.5 | 804.5 | 481, 507 | PE 18:0_18:1(n-10) |
|  |  |  | 495, 521 | PE 18:0_18:1(n-9) |
|  |  |  | 523, 549 | PE 18:0_18:1(n-7) |
|  | 758.5 | 816.5 | 507, 533 | PE 18:1(n-9)_19:1 |
|  |  |  | 535, 561 | PE 18:1(n-7)_19:1 |
|  | 760.6 | 818.5 | 509, 535 | PE 18:1(n-9)_19:0 |
|  |  |  | 537, 563 | PE 18:1(n-7)_19:0 |
|  | 772.5 | 830.5 | 521, 547 | PE 18:1(n-9)_20:1 |
|  |  |  | 549, 575 | PE 18:1(n-7)_20:1 |
|  | 774.5 | 832.5 | 523, 549 | PE 18:1(n-9)_20:0 |
|  |  |  | 551, 577 | PE 18:1(n-7)_20:0 |
|  | 800.5 | 858.5 | 549, 575 | PE 18:1(n-9)_22:1 |
|  |  |  | 577, 603 | PE 18:1(n-7)_22:1 |
| PS | 762.5 | 820.5 | 467, 493 | PS 16:0_18:1(n-9) |
|  |  |  | 495, 521 | PS 16:0_18:1(n-7) |
|  | 788.5 | 846.5 | 493, 519 | PS 18:1(n-9)_18:1(n-9) |
|  |  |  | 521, 547 | PS 18:1(n-7)_18:1(n-7) |
|  | 790.5 | 848.5 | 495, 521 | PS 18:0_18:1(n-9) |
|  |  |  | 523, 549 | PS 18:0_18:1(n-7) |
| **Lipid**  **Subclass** | **[M-H]- *m/z*** | **PB precursor *m/z*** | **Diagnostic ions**  ***m/z*** | **C=C location Isomers** |
| FA | 281.2 | 339.2 | 179, 197 | FA 18:1(n-9) |
|  |  |  | 199, 225 | FA 18:1(n-7) |
